# BRIGHT Coaching: A Randomized Controlled Trial on the Effectiveness of a Developmental Coach System to Empower Families of Children With Emerging Developmental Delay

**DOI:** 10.3389/fped.2019.00332

**Published:** 2019-08-07

**Authors:** Annette Majnemer, Maureen O'Donnell, Tatiana Ogourtsova, Bahar Kasaai, Marilyn Ballantyne, Eyal Cohen, Jean-Paul Collet, Tammie Dewan, Mayada Elsabbagh, Ana Hanlon-Dearman, Jillian Helen Filliter, Lucyna Lach, Theresa McElroy, Patrick McGrath, William McKellin, Anton Miller, Hema Patel, Gina Rempel, Michael Shevell, Kristy Wittmeier, The Parent-Panel

**Affiliations:** ^1^Faculty of Medicine, School of Physical and Occupational Therapy, McGill University, Montreal, QC, Canada; ^2^Montreal Children's Hospital, Research Institute of the McGill University Health Center, Montreal, QC, Canada; ^3^Department of Pediatrics, Faculty of Medicine, University of British Columbia, Vancouver, BC, Canada; ^4^Child-Health BC, Vancouver, BC, Canada; ^5^Holland Bloorview Kids Rehabilitation Hospital, Toronto, ON, Canada; ^6^Hospital for Sick Children, Toronto, ON, Canada; ^7^BC Children's Hospital, Vancouver, BC, Canada; ^8^Department of Pediatrics and Child Health, University of Manitoba, Winnipeg, MB, Canada; ^9^IWK Health Centre, Halifax, NS, Canada; ^10^Department of Pediatrics, Dalhousie University, Halifax, NS, Canada; ^11^McGill University Health Centre, Montreal, QC, Canada; ^12^Department of Occupational Science and Occupational Therapy, Faculty of Medicine, University of British Columbia, Vancouver, BC, Canada; ^13^Rare Disease Foundation, Vancouver, BC, Canada; ^14^Department of Anthropology, University of British Columbia, Vancouver, BC, Canada; ^15^Child and Family Research Institute, Vancouver, BC, Canada; ^16^Children's Hospital Winnipeg, Winnipeg, MB, Canada; ^17^University of Manitoba, Winnipeg, MB, Canada; ^18^Winnipeg Health Sciences Centre, Winnipeg, MB, Canada; ^19^Children's Hospital Research Institute of Manitoba, Winnipeg, MB, Canada

**Keywords:** health coaching, coaching, childhood disability, parents, family, developmental delay

## Abstract

**Background:** In preschool-aged children with, or at elevated risk for, developmental disabilities, challenges and needs arise from vulnerabilities linked to critical and newly emerging cognitive, speech, motor, behavioral, and social skills. For families, this can be a stressful period as they witness the gradual unfolding of their child's differences and await to receive care. Nationally and internationally, service delivery models during this critical period are not standardized nor are they nimble or sufficient enough, leading to long wait times, service gaps and duplications. Given these struggles, there is a need to examine whether “health coaching”, a structured educational program that is deliverable by different and more accessible means, can be effective in empowering families, by delivering information, providing social supports, and decreasing the demands on the overwhelmed health and developmental services. The primary objective is to evaluate the feasibility and the effectiveness of a coaching intervention (in comparison to usual and locally available care), for parents of children with emerging developmental delays.

**Method/Design:** A multi-centered pragmatic randomized controlled trial design will be used. Families will be recruited from a representative sample of those awaiting publicly-funded regional child health services for children with developmental delays in four Canadian provinces. The target sample size is 392 families with children aged 1.5 to 4.5 years at recruitment date. Families will be randomly assigned to receive either the BRIGHT Coaching intervention (coach supported, hardcopy and online self-managed educational resources: 14 sessions, 2 sessions every 4 weeks for 6–9 months) or usual care that is locally available. In addition to the feasibility and acceptability measures, outcomes related to family empowerment, parental satisfaction and efficacy with caregiver competency will be evaluated at baseline, post-treatment (8 months), and follow-up (12 months).

**Discussion:** This manuscript presents the background information, design, description of the interventions and of the protocol for the randomized controlled trial on the effectiveness of BRIGHT Coaching intervention for families of children with emerging developmental delays.

**Trial Registration:** ClinicalTrials.gov, U.S. National Library of Medicine, National Institutes of Health #NCT03880383, 03/15/2019. Retrospectively registered.

## Background

Developmental disabilities, resulting from disorders of the developing nervous system, include limitations in function that begin to manifest during infancy or childhood as delays in reaching developmental milestones or as lack of function in one or multiple areas of cognition, motor performance, vision, hearing and speech, and behavior (1). Children with, or at elevated risk for, developmental disabilities experience chronic lifelong functional consequences with new challenges emerging at each stage of development. In the preschool years (3–6 years), needs arise from vulnerabilities linked to critical and newly emerging cognitive, speech, motor, behavioral, and social skills (2, 3). For families, this can be a stressful period as they witness their child's differences and await assistance to organize health and educational services and receive relevant and appropriate care (i.e., diagnosis and therapeutic interventions) and they must consider the best options in preparation for optimal school entry. Nationally and internationally, service delivery models during this critical period are not standardized (4), and differ considerably within and across jurisdictions and across patient conditions, leading to long wait times, service gaps and duplications (5, 6).

Recently, a focus on care coordination (e.g., care planning, navigating the health-care system) has emerged in the literature for those with chronic diseases (7). At the same time, science has emerged demonstrating how patient education programs that promote self-management for those with specific chronic conditions improve health behaviors, enhance health status, and decrease health-care costs (8). For the most part, health coaching research to date has focused on improving motivation and adherence to health behaviors and to support lifestyle changes in order to prevent the negative consequences of a disease (9). Health coaching is tailored to the patients' knowledge needs, and can be delivered through various, often more accessible means—via technology platforms (10).

Given the aforementioned struggles of affected families in this period of their child's emerging disability and the limitations of the current health-care service delivery models in meeting child and family needs in a timely manner, there is a need for new models of care and support. Specifically, a health coach style of intervention coupled with parent education and peer support delivered through an online platform may be effective in empowering families, by delivering relevant, time-sensitive information, providing social (parent to parent) supports, and decreasing demands on health and developmental services that are often overwhelmed. This method provides an appreciable service re-design in a system at a critical point of transition in child's development.

The primary objective is to evaluate the feasibility and effectiveness of a coaching intervention, utilizing a multi-site pragmatic randomized clinical trial that compares this intervention to usual and locally available care for parents of children with emerging developmental delays.

We hypothesize the following:

1. A standardized approach to coaching (i.e., coach, online education tools, and peer support network) is *feasible* in the real-life context and *acceptable* to caregivers and can be delivered across multiple inter-provincial sites recruiting families from urban/suburban/rural settings.
2. A standardized approach to coaching enhances *parental health* (parents' empowerment and sense of competence, quality of life, and minimizes parenting stress), *family health care experience* (care coordination experience and process of care) at similar or reduced *health care cost* (economic analysis), when compared to usual and locally available care.
3. Given gender-related differences that often exist in caregiver's roles and responsibilities, fathers will contact the coach and use the online platform less frequently than mothers.
4. Parents will use the online platform most frequently during the expansion of referral to and contacts with health professionals in the health care system.
5. During coaching, the participating family's network will move from the professional, health services support to more community, educational and peer support.

## Methods

### Trial Design

This is a prospective, two-arm pragmatic randomized controlled trial (RCT) comparing a coaching and e-health intervention plus usual local care to the control state in which children and their families receive only usual local care over a 12-month time frame. In year 1 (2017-2018), prior to the RCT launch, a technology-supported health coaching service delivery model was developed and refined in conjunction with parents of children with needs similar to those of our study population and a comprehensive needs assessment (refer to *Intervention Development* section for details). In addition, the participating coaches were trained in delivering the intervention using standardized methods. In year 2 (2018–2019), a feasibility/acceptability pilot study was conducted to ensure that the intervention can be delivered in the real-life context across the four participating Canadian provinces. Moreover, during this pilot phase, the fidelity of the coaches in providing the coaching intervention was ascertained.

### Study Setting

The RCT will be conducted within four Canadian provinces (British Columbia, Manitoba, Quebec, and Nova Scotia) and represent the diversity of their urban and rural settings and health systems, including hospitals, clinics and specialized child development centers.

### Eligibility Criteria

The target population is families with children aged 1.5 to 4.5 years old who are referred for diagnosis and/or therapeutic interventions due to emerging delays in one or more domains (e.g., motor, cognitive, speech, social and/or behavioral). Inclusion criteria covers families with: a child placed on a wait list for developmental services or assessments within the past 6 months who is not starting kindergarten within the next 6 months; willingness to complete the three research assessments (baseline, post-intervention, follow-up); regular access to the Internet using a desktop, laptop, or a mobile device; comfort talking and reading in English or in French (for Quebec sites).

### Sample Size

Sample size was calculated with G^*^Power Version 3.1.9.2 (11), while considering a 2 [group: intervention, control] x 2 [time: baseline to post-treatment, post-treatment to follow-up) x 4 [province: British Columbia, Manitoba, Quebec, Nova Scotia] mixed model analysis of variance (2×2×4 ANOVA) with the main outcome measure (i.e., the Family Empowerment Scale, continuous variable) as the dependent variable. Sample size estimates are based on (i) two-sided test of the null hypothesis at α = 0.05, (ii) β = 0.80, (iii) 10% attrition; (iv) a difference of 0.3 standard deviation (SD) on the main outcome measure. Former studies have not identified a minimal clinically important difference (MCID) for our primary outcome measure that could be used to estimate sample size calculation. In the absence of a clinimetric MCID, a distribution-based methods approach is commonly applied that proposes a fraction of the pooled SD (i.e., using effect size estimates). In this case, the difference of 0.3 SD can be used to detect modest effects that may be clinically significant; or 0.5 SD for moderate to large effects (12, 13).

We aim to recruit a sample of 352 participants based on the sample size estimations above (randomized to either intervention or control group) across the four sites. This will be approximately 100 participants from British Columbia, 100 from Quebec, 75 from Manitoba and 75 from Nova Scotia. In order to be able to detect modest effects that may be clinically important (0.3 SD), 392 participants would be needed. Accounting for 10% attrition, a final sample of 352 participants was determined. Furthermore, this larger sample size would enable us to account for cluster randomization. We expect that all participants are independent, regardless of province in which they were recruited. Nonetheless, it is conceivable that the association within provinces is slightly stronger (weak ICC = 0.100) than the association between participants across provinces, due to environmental context (i.e., somewhat different health care and social service systems). A sample size of 346 (recruit 384, 10% attrition) would allow us to account for cluster randomization.

### Study Procedures

#### Recruitment and Randomization

Recruitment will begin with family contact. Each site will manage their own recruitment process and protocol. Eligible families may be referred and recruited to the study through social media channels or referral from the general community. However, most families will be contacted via the center to which they were referred for developmental diagnosis and assessment and/or therapeutic interventions. A member of the clinical team will ask if they are interested to learn more about the BRIGHT Coaching study. If interested, the families' contact information will be shared with the local research assistant (RA) overseeing the trial. The RA will speak to a parent by phone to explain the procedures of the study and follow up with an introductory letter by mail and/or email including a study brochure and the consent form. After 1 to 10 days, the RA will call the family to determine interest in participating and to answer any questions. If interested in participating, the parent can either (1) sign and send the written consent form by mail/email, or (2) agree to a verbal consent process with the RA over the phone (in accordance with the Tri-Council Policy Statement on verbal consent). Participants can terminate their involvement in the study at any point in time.

Ethical approval was obtained from the local Research Ethics Board at each recruitment site. Once consent is provided, participants will complete baseline assessments including documentation of their current health care services and social networks. They will be randomized using a computer-generated algorithm (i.e., concealed allocation); randomization will be stratified by site. The value in cluster randomization to regions (i.e., provinces) was determined to be valuable as access to health-care services, nature of usual care provided, and navigation systems differ considerably across provinces and could present an important confounder effect. The allocation ratio for intervention or control will be 1:1 for each site. Given that this is a pragmatic trial that is meant to be generalized to the real-world context, blinding (assessor, coach, participant) is not appropriate or possible. Outcome measures are primarily self-report in nature, again replicating what would be cost-effective and patient-oriented in the real-world setting.

To minimize bias and possible contamination, the coaches are not working in health-care facilities in which we are recruiting participants. The coaches working with the participants from the treatment group are not in contact with the control participants until the end of the study. The intervention is not described in enough detail anywhere online or in this publication (e.g., topics are only listed without detailed description of the presented material) for the parents who are in the control group to be able to apply it and all of the coaching material and the parent working manuals are password-protected.

#### Development of the Intervention Protocol

The BRIGHT Coaching intervention was developed in over a two-year period (2016-2018). To initiate this process, a national consensus workshop (August 2016, Vancouver, BC) was held, involving forty (*n* = 40) individuals representing a variety of stakeholders (including patient-partners) with an interest or expertise in coaching models and/or childhood disability. This workshop aimed to identify terms and different types of coaching interventions as well as the “key ingredients.” This process guided the team to determine the type of the coaching model that would be most applicable. A second workgroup (August 2017, Vancouver, BC), involving eight (n = 8) team members (co-investigators, coaching exerts, patient-partners), focused on collaborative development of the BRIGHT Coaching themes and its contents. An ensuing standardized manual was developed, including definition of the roles and functions and active elements to successful coaching. Several themes emerged as content areas that would promote family knowledge and competency when systematically addressed through a coaching approach. This collaborative creation process also informed the questions for the online needs assessment survey that followed.

### Online Needs Assessment

An online needs assessment survey was deployed to Canadian families using snowball sampling. The purpose was to influence/inform the content of the coaching intervention and to determine the outcomes and priorities of greatest interest to families. This questionnaire consisted of 13 questions (5- and 4-point Likert-type scale and open-ended questions) and was designed in collaboration with our research team and the parent advisory panel (Appendix 1 in Supplementary Material). Questions were related to the design and logistics of the coaching intervention (e.g., time, sequence of topics), the content of the intervention (e.g., understanding the health care system, promoting child development, promoting social and emotional support), and current trends in seeking information/peer support. The target population was parents residing in Canada (no gender/sex, ethnicity restrictions) whose children (no age, diagnosis restriction) had previously been referred and/or awaited assessment for a developmental delay (or who are presently going through this process).

One hundred and seventy (*n* = 170) parents responded, and one hundred and fifty-two (*n* = 152) answered all questions. On average, responders were parents of children aged 7.8 ± 5.7 years old (median: 6.0; range: 0.5–29 years). Predominantly, the diagnosis or the development delay was reported to be (in descending order): autism spectrum disorder/Asperger's (70.8%); multiple diagnoses (19.1%); attention deficit and hyperactivity disorder (13.4%); global developmental delay (10.1%); speech delay (8.9); cerebral palsy (6.7%); Trisomy 21 (5.6%); other (e.g., learning disability, developmental coordination disorder, sensory/auditory processing disorders: 1–3%).

**Figure 1:**
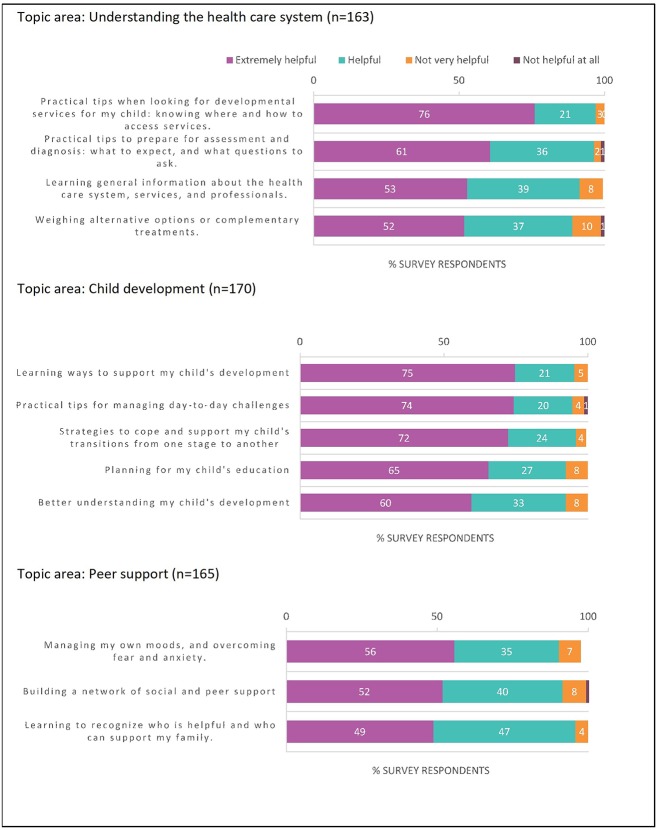
Prevalence of responses for each component within 3 topic areas.

Survey respondents indicated that a reasonable amount of time (per session) to talk to a coach on the phone was (in descending order): 1–2 h (38% of respondents); 2–3 h (31%); 3 or more hours (23%). Figure 1 outlines respondents' interest in covering different topics during the coaching intervention: (1) navigation of the health-care system (where 60.2 ± 11.1% and 33.2 ± 8.2% of respondents report it to be an *Extremely Helpful* and a *Helpful* topic, respectively); (2) child development (where 69.2 ± 6.4 and 25 ± 5.2% of respondents report it to be an *Extremely Helpful* and a *Helpful* topic, respectively); and (3) peer support/personal resilience and health (where 52.3 ± 3.5% and 40.6 ± 6.2% respondents report it to be an *Extremely Helpful* and a *Helpful* topic, respectively). Respondents predominantly considered the presented topics as *Extremely Helpful* to *Helpful*. Moreover, 75% of respondents indicated that they turn to other parents to share experiences when seeking information about their child's developmental needs. In addition, 23–70% reported using online resources (social media, educational websites) to find information to help them with their child's care.

Parent-respondents were also invited to share additional suggestions for the content of the coaching intervention (e.g., other topics, priorities, knowledge gaps, sources of information) beyond what was presented in the survey. Their qualitative responses to the open-ended questions were then analyzed thematically and the following themes emerged: child development-parenting skillset, services and information navigation, advocacy skills (e.g., how to prepare for appointments, questions for the health care professional), prognosis and developmental trajectories, practical suggestions for everyday management, supporting mental and physical health (parent and child), optimizing family dynamics & supports, finances (e.g., financial supports, navigation of publicly-funding sources, disability tax credits), general grievances, current health-care system failings, transition to adulthood, and access to coaching.

Key themes are outlined with salient respondent quotes:

Theme – **Access to coaching** –

“*Having access to a health coach would have been life-changing. I got really burned out trying to research/advocate by myself. My health suffered, and I was less effective advocate for my child. Love the idea of the health coach!*”

“*The transition period is extremely anxiety filled. A coach would be such an amazing part of the process which could reduce parental anxiety thus reducing child anxiety and improving behaviors and outcomes during a tumultuous time.”*

Theme – **Advocacy** –

“*As parents we all end up being advocates, so learning how to usefully advocate with the government, in schools, in healthcare situations is a skill we all need whether we want to or not.”*

Theme – **Child development-parenting skillset** –

“*Sometimes, waiting for a diagnosis can be very long and difficult, focusing on skill, play development tasks help to build parents self-confidence and prepare for diagnosis. Diagnosis is sometimes devastating but having a plan in place with goals already helps families to continue to focus on can instead of can't.”*

Theme – **Family dynamics & support** –

“*Dealing with questions from family and friends. Who to go to for personal support, stress support, marriage support […]?”*

### BRIGHT Coaching Themes

**Figure 2:**
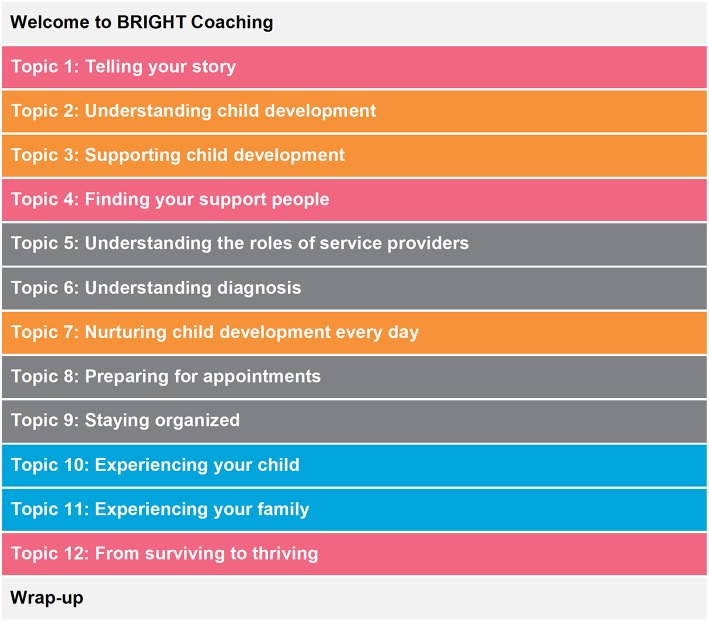
BRIGHT coaching topics and themes.

The design of the coaching intervention was refined as per the preferences and indications of parent-responders to the questionnaire. The parent advisory panel was consulted on multiple occasions, as an iterative process, to assist in interpretation of responses and subsequent intervention design. The final design consisted of fourteen (*n* = 14) themes (Figure 2).

### Coaches' Training and Intervention Manuals

Instructions for specific coaching situations was created based on the assumptions that: (1) caregivers have similar self-management skills and needs; (2) caregivers can develop skills in supporting their child's development, accessing services, and building personal and family resilience; and (3) confident, knowledgeable caregivers will support their children in accessing services that are appropriate and utilize fewer health/social resources if they are more supported in navigating the system.

A registered health professional (i.e., experienced social worker and family counselor with expertise in family/young child counseling) acts as the “Lead Coach,” providing training (multiple formats: individual/group, face-to-face/online), mentoring and oversight of coach activities to ascertain that the intervention is delivered as intended. The coach at each local site has varied backgrounds, but possess the skills required for family support and child development. Three out of four coaches are not registered health professionals (for real-life generalizability and cost purposes). Each coach was trained in and ascertained (by the Lead Coach) to possess skills in motivational interviewing techniques, solution-focused interviewing techniques, individual and collaborative goal-setting and shared decision-making. Coaches received manuals (outlining each topic, with cues/prompts to engage participating parent actively) and on-going training activities (small group discussions, experiential learning and sharing of best practices).

A manual to be used by participating parents during the coaching session was also designed (outlining each topic, workgroup, key concepts, note taking). These manuals were reviewed by the investigators and parent advisory panel to enhance the quality and relevance of the content, as well as to ensure user-friendliness.

### Online Resources and Peer-Support Platform

In addition to the coaching sessions, specific evidence-based educational web content was sourced, vetted and curated by the research team and relevant stakeholders. Input and feedback from a parent advisory group was sought at every stage of development to refine and inform the program content. An online peer-support platform was put in place, where parents can connect with other parents and share their experiences.

## Intervention and Control Treatment

### Intervention

The intervention design is based on three pillars to help guide and support families with a child on a waitlist for services: (1) ***TALK***—to a trained coach while waiting for services/care; (2) ***LEARN***—how to support your child and promote child development via coaching sessions and online resources focused on what to expect and how to be proactive with skill development; and (3) ***SHARE***—connect with other Canadian parents via the online community discussion board where they can share experiences and knowledge.

The coach will call within 1 week of enrolment to provide an overview of the coaching program's goals and content, to enquire about the child's current developmental status. Specific family goals with respect to the parents' involvement in the BRIGHT Coaching program, reflecting their desired needs and services will be set.

The coach will be reachable at least every second day (minimum 4–6 h per day, 3 days per week). Calls will be scheduled at a convenient time for the parent with evening appointments available until 8 pm at least one night per week. Coaching sessions will be done by phone or video calls. A minimum frequency will be defined as 1 coach telephone contact 2 times per month lasting for 45–60 min for each session. The duration of extended contact will be 6–7 months. Intervention will be provided in a flexible manner, as determined by the parents' needs, circumstances and preferences, and the child's developmental condition. Mothers and fathers will both be encouraged to interact with the coach and seek advice and support. Intervention will be discontinued in the event of three missing coaching sessions. Intervention will continue if the participating parent canceled well in advance of the scheduled sessions and has been otherwise engaged with the coach.

The responsibilities of the coach include: (i) guide family in identifying areas of developmental concern (e.g., using the checklist of developmental milestones published by Center of Disease Control & Prevention), (ii) promote developmental stimulation and skills training to optimize development, (iii) support family in addressing their own mental, physical, and family dynamics challenges; and (iv) provide general information regarding the range of developmental services that might be experienced, or of benefit to the child (e.g., seeing an audiologist or neurologist). The coach will aim to cover one topic per call, but may need to continue over a second call, or can cover a second topic on the same call, depending on the knowledge gaps and needs of the parent(s).

The evidence-based educational web content will be hosted on an online care coordination platform—using the *Igloo platform* (https://www.igloosoftware.com/). The team adapted and utilized the *Igloo platform* for this study, placing each family at the center of each network (provincial coach, other participating families, research team), and then allowing each family network to link with other family networks in the intervention arm of the study. This will enable families the opportunity to find and share resources, create connections with the coach and other families, thereby creating a particular network of individuals to support them formally and informally (Appendix 2 in Supplementary Material).

Peer support (among parents in the intervention arm) will be a critical part of this integrated talk-learn-share service delivery model, enabling parents to share strategies. This password-protected website will include links to credible sites based on the family-based needs assessment described above, as well as links to provincial resources relevant to families of children with developmental challenges.

### Control

Families randomized to the control group will receive usual and locally available care. Usual care is highly variable across participating provinces and individual experiences. However, it mainly consists of (i) waiting to access developmental services, including assessment, diagnosis, therapy and/or intervention in a hospital or clinic in their province, (ii) varied intervention approaches offered in terms of type, frequency and location, and/or (iii) private therapies for a subset. Families in the control arm will be contacted at recruitment for baseline information and will be assessed at 8 and 12 months using the outcome measures outlined below. Upon completion of the study, they will be provided with up to three phone sessions with the coach on intervention topics of the family's choosing, as well as providing them with access to the Igloo platform (password-protected site containing the full educational web-content and the online peer support, connecting individuals to others in the control group).

## Outcome Measures

For all participants, the following attributes will be measured at recruitment to characterize the sample: (i) child's sex/gender and age; (ii) first three letters of the postal code and parents' sociodemographic variables (education level, living area (rural/urban), family structure); (iii) child's functional levels using the Vineland Adaptive Behavior Rating Scale 3.0 (14) (phone interview by a level B qualified assessor) not otherwise involved in the trial); (iv) parental stress [Parenting Stress Index (15)]; and (v) developmental services used. Moreover, participants' readiness and willingness to receive coaching will be assessed via an in-house 8-question online survey.

Outcome measures will be conducted at baseline (pre-intervention), 8 months (post-intervention) and 12 months post-entry (follow-up) by the RAs in each province. Each assessment session is expected to last 2– 3 h and participants will receive a $50 gift card following each evaluation visit. The initial outcome (Hypothesis 1) relates to the assessment of feasibility and initial acceptability of the protocol implementation. Protocol feasibility will be evaluated with respect to the ability to recruit and implement a standardized approach to coaching and use of the platform across the four diverse provincial sites. The feasibility and acceptability of the intervention will be evaluated by: percent of successful virtual visits with the coach (defined as the ability to connect with families at home via telephone or a free videoconferencing tool) and completion of the coaching program; participants' utilization of Igloo-based online education and online peer support; parental (mother AND father, if both involved, or other caregiver, as appropriate) satisfaction surveys; and, feedback from the coaches.

The primary outcome of interest (Hypothesis 2) in this pragmatic RCT refers to the *parents' ability to self-manage and promote their child's development*. Thus, the primary outcome measure is the Family Empowerment Scale, which focuses on empowerment at the family (managing the day to day), services (working with the system to receive adequate services) and community (finding or advocating for needed supports, policies, agencies) levels (16). Its psychometric properties are established (17). The Parents' Sense of Competence Scale, measuring satisfaction (anxiety, motivation, frustration) and efficacy (capability, problem-solving) with parental roles will also be administered (18).

Secondary measures of intervention efficacy map onto domains within the Triple Aim Framework (better health, better health care, better value) and will include:

1. Parental well-being: Short-Form 36 (19) (health-related quality of life) and the Parenting Stress Index (15) (parent stress level).
2. Family health care experience: Measure of Process of Care (20) (the extent to which care is family-centered)
3. Health care costs/ service utilization patterns: Resource Utilization Questionnaire (21) (standardized metric for the evaluation of costs of health care and out-of-pocket).
4. Care and social networks that will be collected from a phone-administered survey of network of services.
5. Engagement of mothers and fathers with BRIGHT Coaching program through the Igloo platform: Analysis of online relationships and contact data of participants with their provincial coach and other families assigned to the same intervention arm.

Exploratory outcome measures will include:

1. Child's functional abilities and independence: Vineland Adaptive Behavior Rating Scale – Communication, daily living skills, socialization, and motor skills (22).
2. Acceptability of the BRIGHT coaching program: exit interviews with intervention arm participants at study completion. Content will include key ingredients to the intervention (what they most and least appreciated) and the factors that influenced (positively and negatively) the success of the intervention. Similarly, exit interviews with the coaches will be completed to determine the feasibility and value of the intervention from their perspective.
3. The contacts with the coach will be audiotaped and reviewed by the Lead Coach (clinical expert) to ensure program quality and improvement on an iterative basis.

### Data Collection: Team and Management

Each of the four sites has a part-time RA, with a Study Coordinator at the Montreal site. The national Coordinator will organize regular teleconferences with the provincial RAs to troubleshoot any challenges in recruitment or data collection and will ensure standardization of procedures.

There will be a data sharing agreement signed by the four sites, with common data storage in a secure environment on *REDCap*. This password-protected database will have personal information de-identified and a separate list kept of participants (child, parents) names, birth date of the child, and address/email. The Study Coordinator will have oversight of the database and ensure that there are no missing values.

### Data Analysis

Data will be analyzed at the Data Coordinating Center of the CHILD-BRIGHT Strategies for Patient-Oriented Research (SPOR) Network by a professional external to the study (i.e., not involved in protocol design, data collection or treatment administration). Data from each outcome assessment will be collected and summarized for each group and statistical analyses performed in SAS 9.4 (SAS Institute Inc).

Prior to descriptive and primary analysis, the normality of the data distribution will be ascertained for all studied variables using the Shapiro-Wilk test of normality (23) and Levene's test of equality of error variance (24). Descriptive statistics (frequencies, means, standard deviations for normally distributed data; medians and interquartile ranges for non-parametric data) will be used to characterize our sample in the experimental and control groups and to determine any differences in baseline characteristics between groups [*t*-tests (25) for normally distributed data; Mann-Whitney *U* test (26) for nonparametric data].

Principles of intent-to-treat analysis will be applied for primary analysis. Any missing values after treatment will be imputed by carrying the last observation forward, adhering to a conservative assumption with respect to treatment effects. The primary analysis will be to estimate the between-group (intervention vs. control) differences in the change scores from baseline to 8-months (post-treatment) and from 8 to 12-months (follow-up) post-enrollment. This will be evaluated using a 2 [group: intervention, control] x 2 [time: baseline to post-treatment, post-treatment to follow-up) x 4 [province: British Columbia, Manitoba, Quebec, Nova Scotia] mixed model analysis of variance (2 × 2 × 4 ANOVA) with the outcome measure(s) as the dependent variable(s). We will employ different types of models as well as the combined covariance unstructured type as the reference model. The final model will be chosen using the Akaike's Information Criterion, the Bayesian Information Criterion, and the Restricted Maximum Likelihood Ratio Test. The selected model will be further ascertained by evaluating the fit of the data and deviations from model assumptions using Residuals Analysis. Correction for multiple comparisons will be performed using the Kenward-Roger approach as implemented in SAS® (27). Simple effects along with the two- and three-way interaction terms (group x time; group x province; province x time; group x time x province) will be determined. In the event the interaction term is significant, *post-hoc* analyses using pre-determined pairwise comparisons will be carried out.

Within the intervention group, predictors of change scores will be tested to determine the characteristics of children/families that are more likely to be responsive to BRIGHT coaching. Multivariate linear regressions will be conducted with independent variables (parent readiness for coaching score, sociodemographic factors, child's functioning and primary presenting feature(s) [i.e., delay in motor, speech, social communication, cognitive and/or global areas and/or known diagnosis], province, and engagement in the Igloo platform) and the dependent variables (main outcomes of interest). The structural properties of the network (Igloo) will be analyzed using UCINET (28). Network analysis related to patterns of use of the online platform will be correlated with the outcome measures of interest in this study.

A 10% subset (for each province) of randomly selected exit qualitative reports from families and coaches will be audiotaped and the verbatim will be transcribed and imported into the NVivo software (QSR International, Australia) for data management. Any French statements will be translated into English following the verbatim transcription using a back-translation method. Triangulation methods will be used for analysis of the data (29). More specifically, one coder will read each transcript to gain a general sense of the content's meaning. The transcript's content will then be analyzed by generating initial codes for all meaningful ideas emerging from the data, using a directed content-based analysis technique (30). Following this, a second person will code the transcript using the coding grid. Codes that emerged from the data during the second coding procedure that could not be categorized using the existing grid will be further discussed among both raters to explore their meaning and/or relationship to other codes, and a consensus will be reached. For the remaining of the exit interviews, to elicit common emerging themes, rapid summary sheets will be completed with key questions and themes checks and adjacent notes where necessary. Descriptive statistics on the emergent themes (frequencies, ranges) will be used to summarize and report on these results.

### Discussion and Dissemination Plan

In this paper, we have presented the background and design for a pragmatic randomized controlled trial comparing a BRIGHT Coaching program to usual care for families of preschool-aged children with emerging developmental delays. In light of: (1) several conceptual innovations, accessibility, and patient/family-centeredness of the newly developed BRIGHT coaching model; (2) the shortcomings of current health care service delivery models and their consequences (e.g., long waiting periods, duplication in services, perceived lack of access to credible, accessible knowledge that empowers families (5, 6); (3) the lack of high quality evidence on the effectiveness of health coaching for parents of children with developmental challenges or disabilities despite the surge in use and application of coaching in clinical settings (31, 32), we believe the time is right to conduct a high-quality clinical trial of this size, scope and nature. We foresee that the results will be widely generalizable and applicable outside the context of the RCT and will contribute to the anticipated shifts in health care service delivery models for families. We anticipate that our approach will have positive impacts on the sense of empowerment and resilience of future families with children presenting with emergent developmental delays. We expect parents to experience greater confidence and competence in navigating the health care system and in promoting their personal and family well-being. In turn, we propose that it could lead to beneficial effects on the child's development and integration in the school environment and functioning during the transition period.

Unique to this trial is the integration of the family voice in the design. Rigorous and highly advantageous patient-oriented strategies were employed through all phases of protocol development and building the intervention from inception. A Parent Advisory Group will continue to be engaged in implementation, analysis, interpretation and the dissemination of findings. Given the use of patient-oriented research methods that align with the Canadian Institutes of Health Research's SPOR initiative (33), we speculate the intervention in this trial will result in a patient/family-centered, holistic approach.

The results of this study will be relevant to children and families, health service administrators, policy makers and providers. As such, a wide-scope knowledge translation (KT) dissemination plan will be put in place for the findings of this trial. In order to determine how best to communicate the results to families a focus group will be conducted to guide strategy (content and approach to KT). Content will focus on family perspective regarding the benefits and challenges of the coaching model and value-added to existing resources and supports in the health care system. Approach to KT may include family councils, childhood disability focused organizations, the media, family-guided web-based materials and providers but will be determined conclusively subsequent to the focus group For policy-makers and health/social service decision-makers, the knowledge translation content for this audience will focus on the nature of the intervention, the key ingredients that showed success, and the cost-utility analysis. The KT approach will involve a policy dialogue hosted in year 5, where policy options for implementation will be presented. A policy paper with associated briefing notes, which can be tailored to each province and relevant ministries, will be created. Lastly, for service providers, the content focus will be on the effectiveness of the intervention and its relation to “usual care.” KT approaches will include peer-reviewed articles; presentation at local, provincial, and national meetings; user-friendly summaries and stories on resource websites (e.g., childhooddisability.ca, CHILD-BRIGHT.ca); as well as webinars for the clinical audience (e.g., childrenshealthcarecanada.ca).

## Data Availability

We do not wish to share our data before all data collection is completed and we have thoroughly analyzed it. The datasets analyzed following the completion of the proposed study will be available on reasonable request.

## Ethics Statement

The study was approved by the host institution—McGill University Health Centre (MUHC) Research Ethics Board (REB) on 2017-08-04 (Project number: 2017-3159; Local REB number: IRB00010120). Similar REB approvals have been obtained from all the other participating sites (Child Health BC, BC Children's Hospital, UBC; Specialized Services for Children and Youth (SSCY) Centre; Izaak Walton Killam (IWK) Health Centre). Prior to enrollment informed consent will be obtained from each participant.

## Author Contributions

AMa, MO'D, HP, MS, BK, ME, MB, KW, AMi, and TO developed the study protocol and/or sections of the study protocol. ME, MB, TM, AMi, GR, TO, and BK revised the study protocol and/or sections of the study protocol. AMa, TO, and BK drafted the manuscript. All of the authors critically revised the manuscript for important intellectual content, gave approval of the version to be published and agreed to be accountable for all aspects of the work.

### Conflict of Interest Statement

The authors declare that the research was conducted in the absence of any commercial or financial relationships that could be construed as a potential conflict of interest.

## Abbreviations

ANOVA: Analysis of variance
BC: British Columbia
CIHR: Canadian Institutes of Health Research
KT: Knowledge translation
MCID: minimal clinically important difference
MUHC: McGill University Health Centre
RA: Research assistant
RCT: Randomized Clinical Trial
RI: Research Institute
SAS: Statistical Analysis System
SD: Standard deviation
SPOR: Strategies for Patient Oriented Research
UBC: University of British Columbia.
