# Supplementary material for: BRIGHT Coaching: A Randomized Controlled Trial on the Effectiveness of a Developmental Coach System to Empower Families of Children With Emerging Developmental Delay
Source: Front Pediatr. 2019 Aug 7;7:332. doi: 10.3389/fped.2019.00332 (PMC6694748; doi:10.3389/fped.2019.00332)
Supplement: Supplementary file 1 [file Image_1.pdf]

Appendix 1 Online needs assessment

This survey will help design the coaching program for the main CHILD-BRIGHT study. The families in that main study will have preschool children who are showing one or more delays in development, but they are still waiting to see someone to get an assessment, diagnosis or to access intervention for their child.

We are hoping that coaches will work together with parents to help them feel more confident and competent in their situation. The goal for the coaching is to help parents become informed, to get support, and to talk about topics that are important to families at that stage in their journey. Please think about the time when your child was at the early stages of assessment, diagnosis and or intervention.

In these series of questions, we would like to get your opinion on which topic areas you feel that the coaches can discuss or offer to parents. Please rate each topic below based on how helpful you think it would be for parents to discuss with a developmental coach, while they are in this waiting period. Your opinion will help us design best our study for parents!

1. Child development

	Extremely helpful	Helpful	Not very helpful	Not helpful at all
Better understanding my child's development For example: milestones	○	○	○	○
Learning ways to support my child's development For example: talking to my child, playing games	○	○	○	○
Practical tips for managing day-to-day challenges For example: choosing the right babysitter or daycare	○	○	○	○
Strategies to cope and support my child's transitions from one stage to another. For example: transitioning from home to daycare, or daycare to kindergarten.	○	○	○	○
Planning for my child's education. For example: choosing a school or kindergarten.	○	○	○	○

Please rate each topic below based on how helpful you think it would be for parents to discuss with a developmental coach, while they are in this waiting period.

2. Social support & emotions

	Extremely helpful	Helpful	Not very helpful	Not helpful at all
Building a network of social and peer support.	○	○	○	○
Learning to recognize who is helpful and who can support my family.	○	○	○	○
Managing my own moods, and overcoming fear and anxiety.	○	○	○	○

Please rate each topic below based on how helpful you think it would be for parents to discuss with a developmental coach, while they are in this waiting period.

3. Navigating the health system & services

	Extremely helpful	Helpful	Not very helpful	Not helpful at all
Learning general information about the health care system, services, and professionals.	○	○	○	○
Practical tips when looking for developmental services for my child: knowing where and how to access services.	○	○	○	○
Practical tips to prepare for assessment and diagnosis: what to expect, and what questions to ask.	○	○	○	○
Weighing alternative options or complementary treatments.	○	○	○	○

4. Other topics areas

Are there other important topic areas that are not included here, and that you think would be important to cover between the parents and a developmental coach? If yes, please specify: ____

5. Timing of coaching topic areas

The next series of questions ask you about the order and timing in which general topics should be offered by the coach.

As a parent in this waiting period, which of these general topic areas would you like to have discussed with a developmental coach earlier, rather than later?

	Earlier	Later	Topic should not be covered at all
Child development<br>For example: general information about milestones and stages, practical tips to promote development, planning for education, etc.	◯	◯	◯
Social support & emotions<br>For example: Building a social support system, managing moods and anxiety	◯	◯	◯
Navigating the health system & services<br>For example: practical tips about where to go to for services, how to prepare for your child's assessment date, etc.	◯	◯	◯

6. Other priority topics

Are there are other topics that you think should be covered earlier, or as a priority? If yes, please specify:

____

7. Time spent in coaching

How many hours per month do you think is reasonable for a parent to talk on the phone with a developmental coach?

- ◯ No time at all
- ◯ Less than 1 hour
- ◯ 1-2 hours
- ◯ 2-3 hours
- ◯ 3 or more hours

Since one of the goals of the coaching is to help inform parents about their child, it will be helpful for us to consider how and where parents are getting information from. The next series of questions will help us find out the kinds of resources and websites families currently value and use.

Seeking information and Peer support

8. Which sources of information do you go to about your child's development?

- ☐ My child's pediatrician
- ☐ Another health care professional (e.g. nurse)
- ☐ Social worker
- ☐ My partner
- ☐ Other family members
- ☐ Friends
- ☐ Online support group (e.g. Facebook)
- ☐ Offline support group
- ☐ Other - please specify below

(Please check all that apply)

8. b) If you selected other source of information, please specify which:

____

9. What kind of social media or websites do you use for information to help you with your child's care?

- ☐ Facebook - family & friends (inner circle)
- ☐ Facebook - online support group(s)
- ☐ Twitter
- ☐ Online support group for my child's development
- ☐ Online websites or resources on child care
- ☐ I do not use social media for information about my child's care
- ☐ Other - please specify below

(Please check all that apply)

9. b) If you selected other social media or websites, please specify which:

____

10. What type of information do you look for on these sites?

- ☐ Information about child care
- ☐ Information about educational services
- ☐ Information about social services
- ☐ Medical information about diagnoses
- ☐ Information about rehabilitation programs
- ☐ Information to support parents
- ☐ Information from other parents about their experiences
- ☐ Other - please specify which below.

(Please check all that apply)

10. b) If you selected other type of information, please specify which:

____

A little more about you...

Last page! Thank you for your patience!

11. What is your child's current age (in years)?

____

12. What was your child's suspected developmental delay or diagnosis ? (Optional)

____

13. Please share anything else you feel is important in terms of the study, the health coaching, topics, or your experience of this transition period. We want to learn from your experiences and expertise!

____

14. Are you interested to receive e-mails for other surveys or similar CHILD-BRIGHT studies?

- ◯ Yes
- ◯ No

14. b) If you're interested, please share your e-mail with us:

____
