# Supplementary material for: BRIGHT Coaching: A Randomized Controlled Trial on the Effectiveness of a Developmental Coach System to Empower Families of Children With Emerging Developmental Delay
Source: Front Pediatr. 2019 Aug 7;7:332. doi: 10.3389/fped.2019.00332 (PMC6694748; doi:10.3389/fped.2019.00332)
Supplement: Supplementary file 2 [file Image_2.pdf]

Appendix 2 The Igloo Platform

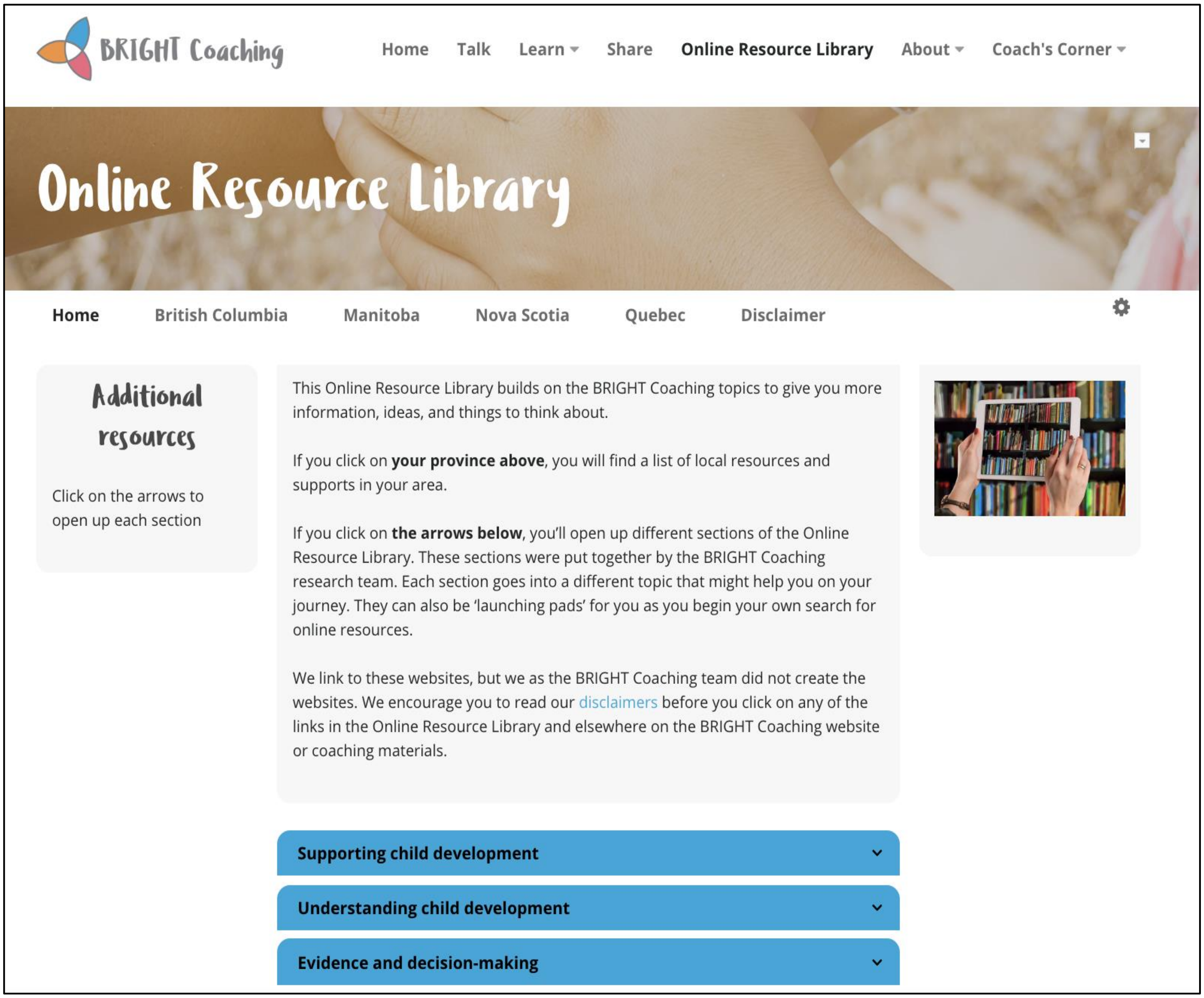

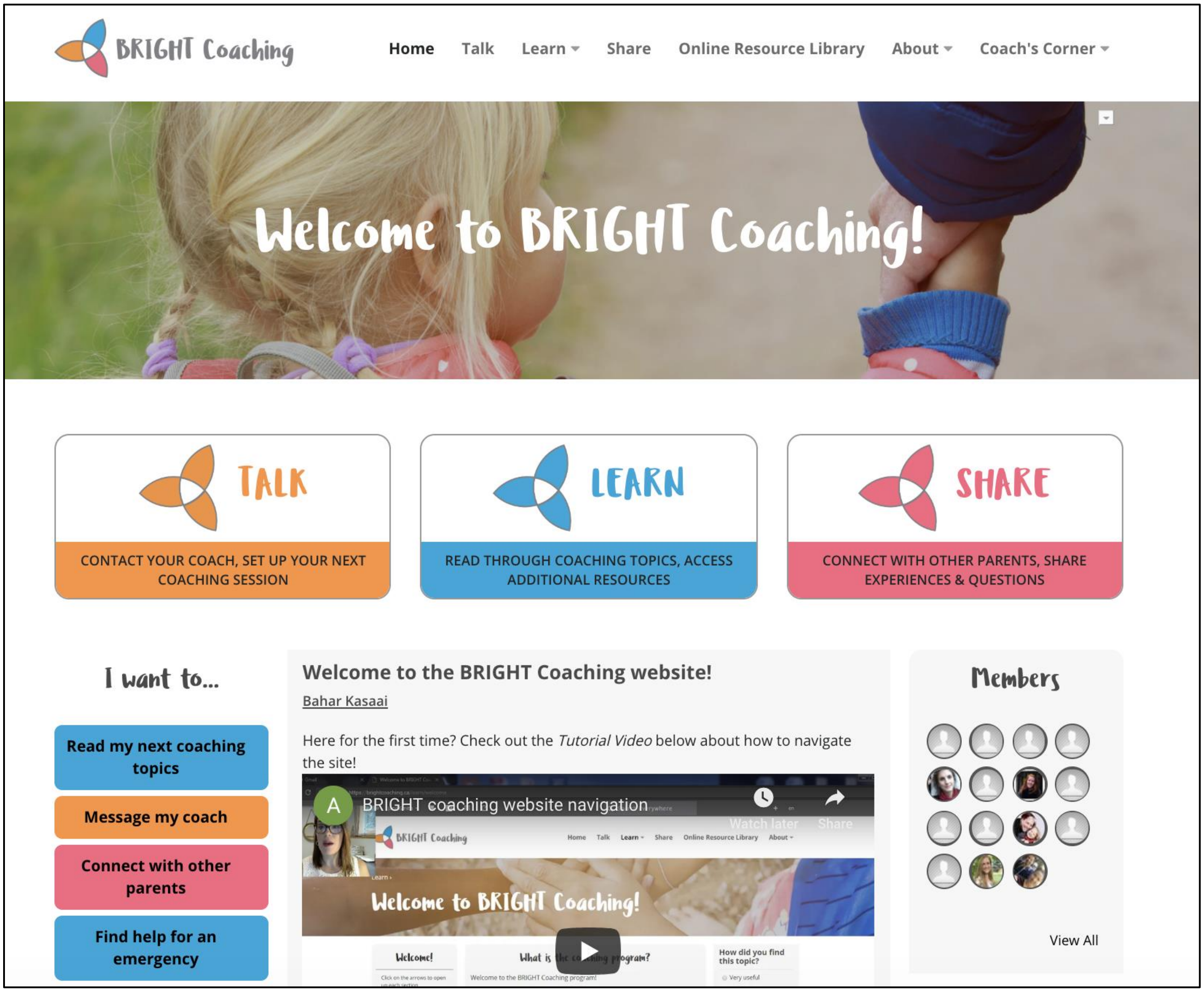
BRIGHT Coaching
Home
Talk
Learn
Share
Online Resource Library
About
Coach's Corner
Welcome to BRIGHT Coaching!
TALK
CONTACT YOUR COACH, SET UP YOUR NEXT COACHING SESSION
LEARN
READ THROUGH COACHING TOPICS, ACCESS ADDITIONAL RESOURCES
SHARE
CONNECT WITH OTHER PARENTS, SHARE EXPERIENCES & QUESTIONS
I want to...
Read my next coaching topics
Message my coach
Connect with other parents
Find help for an emergency
Welcome to the BRIGHT Coaching website!
Bahar Kasaai
Here for the first time? Check out the Tutorial Video below about how to navigate the site!
BRIGHT coaching website navigation
Watch later
Share
Welcome to BRIGHT Coaching!
Welcome!
How did you find this topic?
Members
View All

Topic 2: Understanding child development

Welcome to Topic 2!

Click on the arrows to open up each section

Search in this area

Download PDF

What is this topic about?

What does 'child development' mean?
This topic will help you further develop skills to discuss child development with the professionals who will be providing care and services for your child. It is part of the *'promoting child development'* theme.

Your child's service providers (including doctors, nurses, social workers, therapists, or other professionals) use a certain language to describe child development. Knowing this language will help you talk with these service providers. It can even help you get the right services and care for your child.

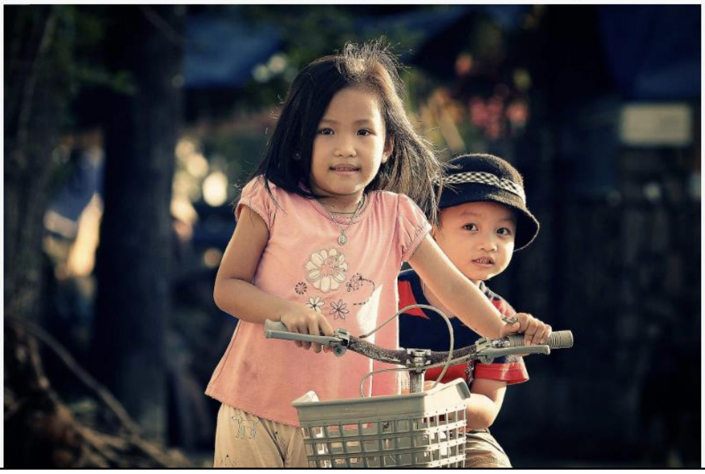

How did you find this topic?

- Very useful
- Somewhat useful
- Not very useful

Vote View Results

1

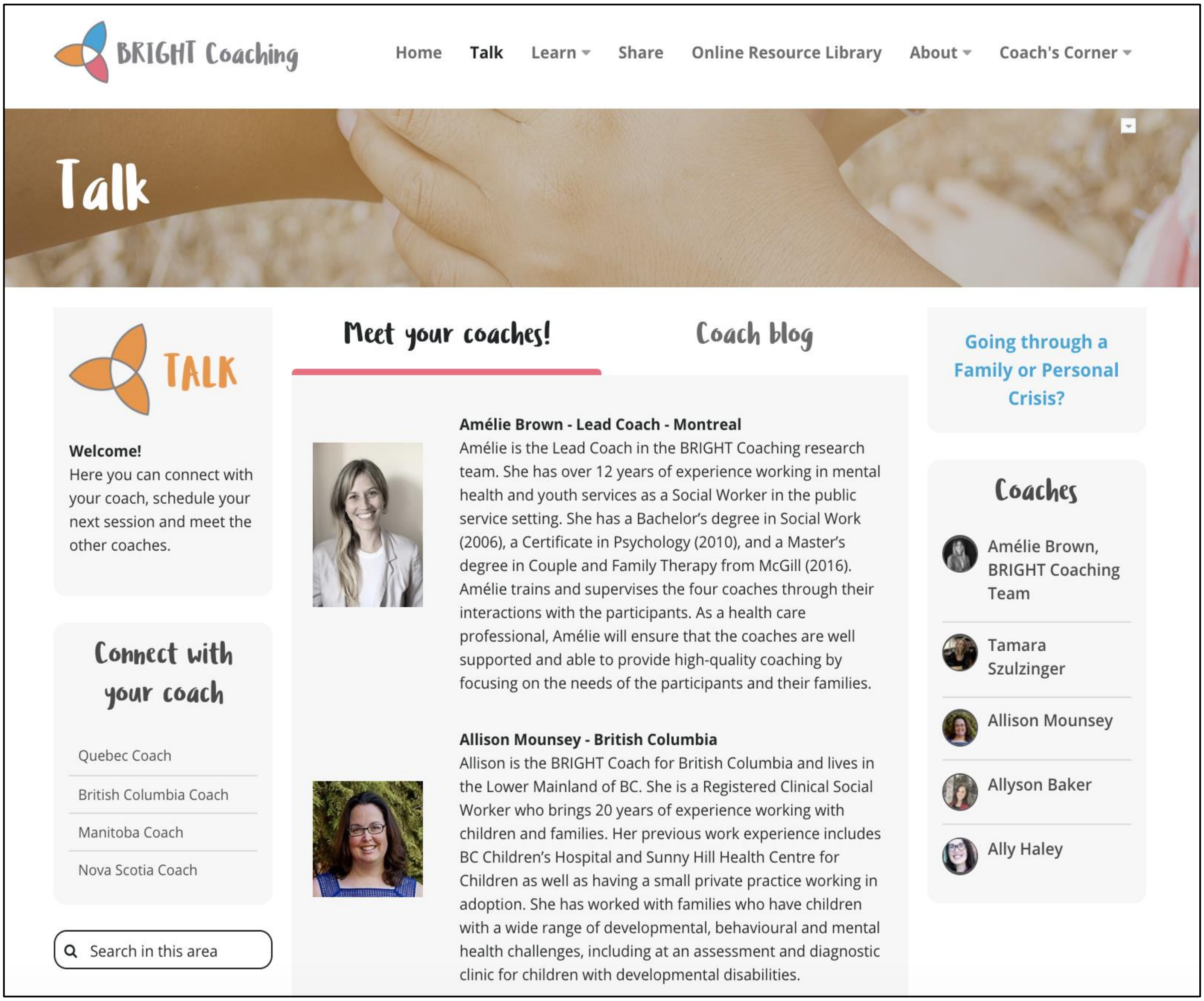
BRIGHT Coaching
Home
Talk
Learn
Share
Online Resource Library
About
Coach's Corner
Talk
TALK
Welcome!
Here you can connect with your coach, schedule your next session and meet the other coaches.
Connect with your coach
Quebec Coach
British Columbia Coach
Manitoba Coach
Nova Scotia Coach
Search in this area
Meet your coaches!
Coach blog
Amélie Brown - Lead Coach - Montreal
Amélie is the Lead Coach in the BRIGHT Coaching research team. She has over 12 years of experience working in mental health and youth services as a Social Worker in the public service setting. She has a Bachelor's degree in Social Work (2006), a Certificate in Psychology (2010), and a Master's degree in Couple and Family Therapy from McGill (2016). Amélie trains and supervises the four coaches through their interactions with the participants. As a health care professional, Amélie will ensure that the coaches are well supported and able to provide high-quality coaching by focusing on the needs of the participants and their families.
Allison Mounsey - British Columbia
Allison is the BRIGHT Coach for British Columbia and lives in the Lower Mainland of BC. She is a Registered Clinical Social Worker who brings 20 years of experience working with children and families. Her previous work experience includes BC Children's Hospital and Sunny Hill Health Centre for Children as well as having a small private practice working in adoption. She has worked with families who have children with a wide range of developmental, behavioural and mental health challenges, including at an assessment and diagnostic clinic for children with developmental disabilities.
Going through a Family or Personal Crisis?
Coaches
Amélie Brown, BRIGHT Coaching Team
Tamara Szulzinger
Allison Mounsey
Allyson Baker
Ally Haley
